# Mental Health and Psychosocial Risk and Protective Factors Among Black and Latinx Transgender Youth Compared With Peers

**DOI:** 10.1001/jamanetworkopen.2021.3256

**Published:** 2021-03-26

**Authors:** Stanley Ray Vance, Cherrie B. Boyer, David V. Glidden, Jae Sevelius

**Affiliations:** 1 Division of Adolescent and Young Adult Medicine, Department of Pediatrics, University of California, San Francisco; 2 Department of Epidemiology and Biostatistics, University of California, San Francisco; 3 Department of Medicine, Center for AIDS Prevention Studies, University of California, San Francisco

## Abstract

**Importance:**

Black and Latinx transgender youth experience stigma that may increase their susceptibility to mental health symptoms.

**Objective:**

To compare past-year mental health symptoms and psychosocial factors among Black and Latinx transgender youth, White transgender youth, and Black and Latinx cisgender youth.

**Design, Setting, and Participants:**

This survey study used data from the 2015-2017 Biennial California Healthy Kids Survey with a weighted sample (N = 45 269) representative of California’s secondary school population. The analytic sample (n = 19 780) included Black and Latinx transgender youth, White transgender youth, and Black and Latinx cisgender youth in the 9th and 11th grades. Data analysis was conducted from July 2020 to February 2021.

**Main Outcomes and Measures:**

Outcomes include past-year depressive symptoms and suicidality. Psychosocial risk factors include school-based victimization, gender-based harassment, sexuality-based harassment, and race-based harassment; protective factors include school connectedness and caring adult relationships.

**Results:**

The analytic sample of 19 780 participants (in 9th grade: weighted percentage, 51% [95% CI, 50% to 52%]; female participants: weighted percentage, 50% [95% CI, 49% to 51%]) included 252 Black and Latinx transgender youth (weighted percentage, 1.3% [95% CI, 1.1% to 1.5%]), 104 White transgender youth (weighted percentage, 0.7% [95% CI, 0.6% to 0.8%]), and 19 424 Black and Latinx cisgender youth (weighted percentage, 98.0% [95% CI, 97.8% to 98.2%]). For Black and Latinx transgender youth, estimated prevalence of depressive symptoms and suicidality were 50% (95% CI, 44% to 57%) and 46% (95% CI, 39% to 52%), respectively. Logistic regression models adjusted for grade, living arrangement, and reported sex indicated that compared with White transgender youth, Black and Latinx transgender youth had similar odds of depressive symptoms (adjusted odds ratio, 0.6; 95% CI, 0.4 to 1.1) and suicidality (adjusted odds ratio, 1.1; 95% CI, 0.6 to 1.8) and similar odds of all forms of harassment (eg, race-based harassment: adjusted odds ratio, 1.5; 95% CI, 0.8 to 2.6). Regression models indicated that compared with White transgender youth, Black and Latinx transgender youth had similar levels of victimization (adjusted linear regression coefficient, 0.5; 95% CI, −0.3 to 1.3) and caring adult relationships (adjusted linear regression coefficient, −0.6; 95% CI, −1.4 to 0.09) but lower levels of school connectedness (adjusted linear regression coefficient, −1.6; 95% CI, −2.9 to −0.4). With similar analyses, compared with Black and Latinx cisgender youth, Black and Latinx transgender youth had higher odds of depressive symptoms (adjusted odds ratio, 2.7; 95% CI, 2.0 to 3.7) and suicidality (adjusted odds ratio, 5.9; 95% CI, 4.3 to 8.0), higher odds and levels of all forms of harassment and victimization (eg, race-based harassment: adjusted odds ratio, 3.2; 95% CI, 2.4 to 4.5), and lower levels of school connectedness (adjusted linear regression coefficient, −2.6; 95% CI −3.3 to −1.8) and caring adult relationships (adjusted linear regression coefficient, −0.9; 95% CI −1.3 to −0.5).

**Conclusions and Relevance:**

In this study, Black and Latinx transgender youth had high rates of mental health symptoms, with rates comparable with White transgender youth but higher than Black and Latinx cisgender youth. Their unique pattern of psychosocial risk and protective factors for these mental health symptoms should be factored in clinical preventive services and school-based interventions to support them.

## Key Points

**Question:**

Do Black and Latinx transgender youth experience greater mental health symptoms than their White transgender and Black and Latinx cisgender peers?

**Findings:**

In this survey study of 19 780 participants, analyses of a statewide school-based survey data set indicated that Black and Latinx transgender youth had high rates of depressive symptoms and suicidality, similar to those among White transgender youth but higher than those among Black and Latinx cisgender youth.

**Meaning:**

These findings suggest that Black and Latinx transgender youth are susceptible to mental health symptoms that should be addressed through clinical services that consider both race/ethnicity and gender identity.

## Teaser

This survey study compares past-year mental health symptoms and psychosocial factors among Black and Latinx transgender youth, White transgender youth, and Black and Latinx cisgender youth.

## Introduction

Although Black and Latinx transgender youth (BLTY) are culturally and psychosocially heterogenous, they share experiences of belonging to racial/ethnic minority groups and being gender diverse, meaning their gender identity does not align with the gender assumed from their birth-assigned sex. Having dual-minority realities, they may experience both transphobia and racism, which may amplify their susceptibility to poorer mental health outcomes.^1^ Higher rates of depression and suicidality among the general transgender youth population compared with cisgender (ie, nontransgender) peers are well documented.^2,3,4,5^ Compared with White youth, Black and Latinx youth have higher rates of mood symptoms or disorders while being less likely to have access to mental health services.^6,7,8,9^

There is a paucity of comparable data focused specifically on the mental health of BLTY. One study of past-year suicidality in a representative sample of Californian students^10^ did not find significant differences between White transgender youth and transgender youth from racial/ethnic minority groups; comparisons of other mental health symptoms were not reported. Other quantitative studies have not reported direct comparisons of mental health symptoms between BLTY and other youth cohorts that share lived experiences of gender diversity or racial/ethnic minority status.^2,3,11,12,13,14,15,16^ Black and Latinx transgender adults face a tremendous burden of poor outcomes, including high levels of depression and substance abuse and negative psychosocial experiences, including racial discrimination, transphobia, and physical victimization.^17,18,19,20,21,22,23^ To our knowledge, the youth antecedents to these poor adult outcomes for Black and Latinx transgender individuals have not been elucidated.

School is an important socioecological domain where youth spend a large portion of time and have psychosocial experiences with peers and nonparental adults that influence their mental health.^24,25^ School-based verbal and physical harassment and bullying are prevalent among US students, with higher rates of victimization reported among transgender students.^3^ Research indicates that past-year victimization at school was associated with past-year suicidality among transgender youth.^10^ Perceived harassment due to gender, gender expression, and sexuality has been found to contribute to mental health symptoms among transgender youth.^4,26,27^ In contrast, school-based protective factors, such as school connectedness and caring adult relationships at school, have been found to be protective against mental health symptoms.^4,28,29,30^ To our knowledge, these potential risk and protective factors for mental health symptoms have not been examined specifically among BLTY.

Examining associations between risk and protective factors in the context of school is critical for addressing mental health disparities experienced by BLTY. Using a large, school-based survey sample representative of the California secondary student population, our study addresses 3 specific aims. First, we compare BLTY with White transgender peers and Black and Latinx cisgender peers to document prevalence of past-year depressive symptoms and suicidal ideation. Second, we compare these peer groups to assess differences in psychosocial risk and protective factors. Finally, we examine associations between psychosocial risk and protective factors and mental health symptoms among BLTY. Based on prior research, we hypothesized that BLTY experience disparities in mental health symptoms compared with their peers. Moreover, we posited that mental health symptoms among BLTY would be associated with higher rates of victimization and different types of harassment (ie, gender-based, sexuality-based, and race-based) and fewer protective factors, such as school connectedness and caring adult relationships at school. Although BLTY are a heterogenous group, our overall goal was to provide critically needed documentation of potential disparities in mental health and psychosocial factors given BLTY risk of experiencing different forms of stigma.

## Methods

### Data Source

Data were derived from 9th- and 11th-grade students who participated in the 2015-2017 Biennial State California Healthy Kid Survey.^31^ This survey is a modular, self-reported assessment administered to students biennially by WestEd with support from the California Departments of Education and Health Care Services to comprehensively capture California youth health risk and resilience data. Schools were randomly selected, and student data were weighted to reflect the California secondary school student population; the survey was administered from fall 2015 to spring 2017. In compliance with state law, opt-out (passive) parental consent was required for survey participation. If a parent did not want their child to participate, they could provide written or verbal notification to the school; students had to assent to complete the survey.^31^ Students excluded from the survey included 1.1% of respondents due to questionable response validity, and an additional 1.7% of respondents were excluded due to missing data in required survey modules. The final sample included 45 269 students. In the 2015-2017 survey, 7th-grade students were not asked about suicidal ideation; thus, they were excluded from this research. The California Office of Statewide Health Planning and Development Committee for the Protection of Human Subjects approved survey implementation. The University of California, San Francisco institutional review board approved this study.

### Measures

#### Sociodemographic Measures

A single item assessed both gender identity and sexual orientation: “Which of the following best describes you? (Mark All That Apply.): Heterosexual (straight), Gay or Lesbian or Bisexual, Transgender, Not sure, Decline to respond.” From these options, a dichotomous transgender variable was derived with 0 indicating cisgender for all participants who did not mark transgender and 1 indicating transgender for those who marked transgender. We derived the sexual identity variable into 4 categories: sexual minority if they selected gay, lesbian, or bisexual; heterosexual if they only checked heterosexual; unsure if they only checked not sure; and no answer if they declined to answer. Youth who selected gay, lesbian, or bisexual were categorized as sexual minority even if other answers were selected. Students were queried on their race (“What is your race?”) and Hispanic/Latino origin (yes or no). The race and ethnicity items of interest were combined into 3 mutually exclusive race/ethnicity categories: non-Hispanic/Latinx White, non-Hispanic/Latinx Black, and Hispanic/Latinx. A peer group variable was derived with the following groups: BLTY (inclusive of all transgender students who were categorized as non-Hispanic/Latinx Black and/or Hispanic/Latinx), White transgender youth (non-Hispanic White youth who selected transgender) and Black and Latinx cisgender youth (all non-Hispanic/Latinx Black and Latinx youth who did not select transgender).

#### Mental Health Outcomes

Depressive symptoms were measured using 1 dichotomous question, “During the past 12 months, did you ever feel so sad or hopeless almost every day for 2 weeks or more that you stopped doing some usual activities?” Suicidal ideation was measured using 1 dichotomous question, “During the past 12 months, did you ever seriously consider attempting suicide?”

#### Psychosocial Risk and Protective Factors

School-based victimization was assessed with a 9-item measure. Each item asked whether the participant had experienced the following in the past 12 months: “been pushed, shoved, slapped, hit, or kicked by someone who wasn’t just kidding around”; “been afraid of being beaten up”; “had mean rumors or lies spread about you”; “had sexual jokes, comments, or gestures made to you”; “been made fun of because of your looks or the way you talk”; “had your property stolen or deliberately damaged, such as your car, clothing or books”; “been threatened or injured with a weapon (gun, knife, club, etc.)”; “been threatened with harm or injury”; and “been made fun of, insulted, or called a name.” Each item’s response was dichotomized to 0 times or 1 or more times, and a count variable was created with score 0 indicating no victimization and 9 indicating high victimization.^10,31^ Harassment was assessed by 3 questions focused on frequency of 3 different types of harassment due to real or perceived belonging to specific social groups. For these types of harassment, students were asked, “During the past 12 months, how many times on school property were you harassed or bullied for any of the following reasons?: Your race, ethnicity, or national origin; Your gender (being male or female); Because you are gay or lesbian or someone thought you were.” Each harassment item’s response was dichotomized to 0 times or 1 or more times and coded as race-based harassment, gender-based harassment, and sexuality-based harassment, respectively.

School connectedness was assessed using a 5-item measure that was calculated by adding the responses to each item.^32,33,34^ An example school connectedness item was “I feel close to people at this school,” with 1 indicating strong disagreement and 5 indicating strong agreement. Caring adult relationship scores were calculated from a 3-item scale for which a composite score was created by summing raw Likert scores.^10,32,33^ An example caring adult item was, “At my school, there is a teacher or some other adult who really cares about me,” with responses of 1 indicating not at all true and 4 indicating very much true.

#### Covariates

Sociodemographic covariates included reported sex, grade, and living arrangement. Reported sex was measured with the question, “What is your sex?” (male or female). Of note, the survey did not provide a definition of sex, sex assigned at birth, or gender identity. For living arrangement, students were asked, “What best describes where you live?” Students could select 1 of 8 categories. The variable was recoded and dichotomized to 1 of the following: a home with at least 1 parent or guardian or an alternative living arrangement (eg, other relative’s home or foster/group care).

### Statistical Analysis

Statistical analyses were conducted starting July 15, 2020 and concluded February 5, 2021. Descriptive and multivariable analyses were conducted in Stata version 16.1 (StataCorp). All analyses used the sampling plan and sampling weights provided by WestEd using the Stata svy package. Descriptive analyses were conducted for demographic characteristics, mental health symptoms, and psychosocial factors. Because the sample was weighted, estimated prevalence was calculated for dichotomous measures, and estimated means were calculated for continuous measures. A series of logistic regression analyses were conducted to compare mental health symptoms and dichotomous psychosocial factors between BLTY and White transgender youth and between BLTY and Black and Latinx cisgender youth and to examine associations between mental health symptoms and psychosocial factors for BLTY. Linear regression analyses were used to compare peer groups on continuous psychosocial factors. All regression analyses included grade, reported sex, and living arrangement as covariates given their associations with mental health symptoms.^35,36,37,38^ All hypothesis testing used 2-tailed *P* < .05 as the significance level and 95% CIs. Cases with missing data were list-wise deleted for all analyses.

## Results

A total of 45 269 students completed the survey, and 566 (1.3%) identified as transgender. Data from a total of 19 780 students were used for this study because they were in 9th or 11th grade and belonged to the derived peer groups, ie, BLTY, White transgender youth, or Black and Latinx cisgender youth. The 19 780 participants (9th grade: weighted percentage, 51% [95% CI, 50%-52%]; female participants: weighted percentage, 50% [95% CI, 49%-51%]) included 252 BLTY (weighted percentage, 1.3% [95% CI, 1.1%-1.5%]), 104 White transgender youth (weighted percentage, 0.7% [95% CI, 0.6%-0.8%]), and 19 424 Black and Latinx cisgender youth (weighted percentage, 98.0% [95% CI, 97.8%-98.2%]). Table 1 presents the demographic characteristics for each peer group.

**Table 1.** Demographic Characteristics for Black and Latinx Transgender Youth and Their Peers

Characteristic	Youth, prevalence, % (95% CI)^a^		
	Transgender		Black and Latinx cisgender (n = 19 424)
	Black and Latinx (n = 252)	White (n = 104)	
Grade			
9th	52 (46-58)	50 (40-59)	51 (50-52)
11th	48 (42-54)	50 (40-60)	49 (48-50)
Sex			
Female	65 (58-70)	61 (51-70)	50 (49-51)
Male	35 (30-42)	39 (30-49)	50 (49-51)
Race/ethnicity^b^			
Non-Hispanic			
White	NA	100	NA
Black	11 (7-18)	NA	10 (9-10)
Hispanic/Latinx	89 (83-93)	NA	90 (90-91)
Sexual identity			
Sexual minority	31 (26-37)	37 (28-46)	3 (2-3)
Heterosexual	24 (19-30)	26 (19-36)	77 (77-78)
Not sure	6 (4-10)	4 1-10)	4 (3-4)
No answer	39 (33-45)	33 (25-43)	16 (16-17)
Alternative living arrangement^c^	4 (2-8)	2 (1-8)	3 (2-3)

Abbreviation: NA, not applicable.

^a^ Estimated prevalence and 95% CIs are presented given weighted sample analyzed.

^b^ Mutually exclusive categories of race and ethnicity were created.

^c^ Alternative living arrangement was defined as any living anywhere besides in a home with at least 1 parent or guardian. Alternative living arrangements include other relative’s home; a home with more than 1 family; a friend’s home; a foster home, group care, or waiting placement; hotel or motel; shelter, care, campground, or other transitional or temporary housing; or other living arrangement.

Table 2 provides prevalence estimates and 95% CIs for past-year depression symptoms, past-year suicidality, and all forms of harassment for each peer group. For BLTY, estimated prevalence of depressive symptoms and suicidality were 50% (95% CI, 44%-57%) and 46% (95% CI, 39%-52%), respectively. Table 2 also provides estimated means for each peer group for the continuous psychosocial factors of victimization, school connectedness, and caring adult relationships. Of note, higher scores for victimization indicate higher victimization, while higher scores for school connectedness and caring adult relationships indicate higher levels of these psychosocial factors.

**Table 2.** Estimated Prevalence and Means of Mental Health Symptoms and Psychosocial Factors by Peer Groups

Measure	Youth					
	Transgender					Black and Latinx cisgender
	Black and Latinx transgender			White transgender		
**Dichotomous measures, estimated prevalence, % (95% CI)**^**a**^						
Depression symptoms	50 (44-57)		63 (53-72)			31 (31-32)
Suicidal ideation	46 (39-52)		47 (38-57)			15 (15-16)
Harassment						
Race-based	33 (27-39)		25(18-33)			13 (12-13)
Gender-based	39 (33-46)		39 (30-48)			6 (6-7)
Sexuality-based	36 (30-42)		45 (35-54)			7 (6-7)
**Continuous measures, estimated mean (95% CI)**^**b**^						
Victimization, range 0-9^c^	3.4 (3.0-3.9)	3.0 (2.4-3.6)			1.7 (1.6-1.7)	
School connectedness, range 5-25^c^	14.6 (13.9-15.3)	15.9 (14.9-16.9)			17.1 (17.0-17.1)	
Caring adult relationship, range 3-12^c^	7.2 (6.8-7.6)	7.8 (7.2-8.4)			8.0 (7.9-8.0)	

^a^ Estimated prevalence for dichotomous measures with 95% CIs are presented given weighted sample analyzed.

^b^ Estimated means for continuous measures with 95% CIs are presented given weighted sample analyzed.

^c^ Higher victimization scores correspond with higher levels of victimization; higher school connectedness scores correspond with higher levels of school connectedness; and higher caring adult relationship scores corresponds to higher levels of caring adult relationships.

Mental health symptoms and psychosocial factors were compared between BLTY and White transgender youth and between BLTY and Black and Latinx cisgender youth (Table 3). There were no significant differences between BLTY and White transgender youth in terms of mental health symptoms (depressive symptoms: adjusted odds ratio, 0.6; 95% CI, 0.4 to 1.1; suicidality: adjusted odds ratio, 1.1; 95% CI, 0.6 to 1.8), victimization (adjusted linear regression coefficient, 0.5; 95% CI, −0.3 to 1.3), and forms of harassment (eg, race-based harassment: adjusted odds ratio, 1.5; 95% CI, 0.8 to 2.6). BLTY and White transgender youth had similar levels caring adult relationships (adjusted linear regression coefficient, −0.6; 95% CI, −1.4 to 0.09), but BLTY had lower levels of school connectedness than White transgender youth (adjusted linear regression coefficient, −1.6; 95% CI, −2.9 to −0.4). Compared with Black and Latinx cisgender youth, BLTY had 2.7 (95% CI, 2.0 to 3.7) higher odds of past-year depressive symptoms and 5.9 (95% CI 4.3 to 8.0) higher odds of past-year suicidality. Additionally, BLTY had higher odds of each form of harassment when compared with Black and Latinx cisgender youth, including race-based harassment (adjusted odds ratio, 3.2; 95% CI, 2.4 to 4.5). BLTY had significantly higher victimization (adjusted linear regression coefficient, 1.8; 95% CI, 1.3 to 2.3) and significantly lower levels of school connectedness (adjusted linear regression coefficient, −2.6; 95% CI −3.3 to −1.8) and caring adult relationships (adjusted linear regression coefficient, −0.9; 95% CI −1.3 to −0.5) compared with Black and Latinx cisgender youth.

**Table 3.** Comparison of Mental Health Symptoms and Psychosocial Factors Between Black and Latinx Transgender Youth and Peer Groups

Measure	Adjusted odds ratio (95% CI)	
	White transgender youth vs Black and Latinx transgender youth	Black and Latinx cisgender youth vs Black and Latinx transgender youth
Dichotomous measures^a^		
Depression symptoms	0.6 (0.4 to 1.1)	2.7 (2.0 to 3.7)^b^
Suicidal ideation	1.1 (0.6 to 1.8)	5.9 (4.3 to 8.0)^b^
Harassment		
Race-based	1.5 (0.8 to 2.6)	3.2 (2.4 to 4.5)^b^
Gender-based	1.2 (0.6 to 2.0)	12.9 (9.3 to 17.9)^b^
Sexuality-based	0.7 (0.4 to 1.2)	7.8 (5.8 to 10.7)^b^
Continuous measures, adjusted linear regression coefficient (95% CI)^c^		
Victimization	0.5 (−0.3 to 1.3)	1.8 (1.3 to 2.3)^d^
School connectedness	−1.6 (−2.9 to −0.4)^d^	−2.6 (−3.3 to −1.8)^d^
Caring adult relationship	−0.6 (−1.4 to 0.09)	−0.9 (−1.3 to −0.5)^d^

^a^ Logistic regression analyses were conducted for dichotomous measures and were adjusted for grade, reported sex, and living arrangement.

^b^ *P* < .05, as indicated by 95% CI not including 1.

^c^ Linear regression analyses were conducted for continuous measures and were adjusted for grade, reported sex, and living arrangement.

^d^ *P* < .05 as indicated by 95% CI not crossing 0.

The multivariable regression models assessing associations between mental health symptoms and psychosocial factors for BLTY appear in Table 4. All forms of harassment and victimization were associated with higher odds of both past-year depression and suicidality among BLTY when controlling for demographic covariates. Neither school connectedness nor caring adult relationships were associated with mental health symptoms among BLTY.

**Table 4.** Association of Psychosocial Factors With Mental Health Symptoms in Black and Latinx Transgender Youth

Factor	Black and Latinx transgender youth, adjusted odds ratios (95% CI)^a^
**Depression symptoms**	
Harassment	
Race-based	2.4 (1.2-4.9)^b^
Gender-based	5.7 (2.7-12.2)^b^
Sexuality-based	7.3 (3.3-16.0)^b^
Victimization	1.3 (1.1-1.4)^b^
School connectedness	1.0 (1.0-1.1)
Caring adult relationship	1.0 (0.9-1.1)
**Suicidality**	
Harassment	
Race-based	3.1 (1.5-6.3)^b^
Gender-based	6.8 (3.3-13.9)^b^
Sexuality-based	7.7 (3.7-15.8)^b^
Victimization	1.3 (1.2-1.5)^b^
School connectedness	1.0 (0.9-1.0)
Caring adult relationship	1.0 (0.9-1.1)

^a^ Logistic regression analyses were conducted and adjusted for grade, reported sex, and living arrangement.

^b^ *P* < .05 as indicated by 95% CI not crossing 1.

## Discussion

To our knowledge, this is the first study of a large population-based sample with a robust number of transgender youth, permitting investigation of differences in mental health symptoms and psychosocial risk and protective factors among BLTY compared with peers. Our approach provided a unique opportunity to compare BLTY with other youth with whom BLTY potentially share experiences of stigma and discrimination, such as harassment and bullying. BLTY were compared with White transgender youth, with whom BLTY may share experiences, such as bullying because of their gender expression or being misgendered (being referred to by incorrect names or pronouns). Additionally, BLTY were compared with Black and Latinx cisgender youth, with whom they share racial/ethnic minority status. Our study elucidates the unique realities and stigma BLTY experience within school settings.

Compared with White transgender youth, BLTY experienced similar odds of mental health symptoms, counter to our initial hypothesis. However, the pattern of psychosocial risk and protective factors were unique for BLTY. For BLTY, victimization and harassment increased their risk of mental health symptoms, but school connectedness and caring adult relationships were not protective. These findings suggest that it may be particularly salient for pediatric clinicians to screen BLTY for school-based bullying and partner with schools to mitigate such harassment.

Consistent with our hypothesis, compared with Black and Latinx cisgender youth, BLTY had significantly higher odds of mental health symptoms, higher odds and levels of psychosocial risk factors, and lower levels of psychosocial protective factors. Despite potentially similar experiences of racism, BLTY had higher odds of experiencing race-based harassment compared with Black and Latinx cisgender youth. This finding may point to the complex interplay of BLTY’s unique experiences with stigma or how BLTY perceive harassment in relation to their identities, including their race. For example, a BLTY student who is seen as different by peers because of both their race and gender identity may experience or perceive more harassment in general. Moreover, our findings suggest that school-based stigma experienced by BLTY affects their mental health. BLTY would benefit from pediatric clinicians evaluating their symptoms while considering stigma stemming from both race and gender identity.

Our study suggests multiple implications. The rates of mental health symptoms for both BLTY and White transgender youth are notably higher than estimates for transgender students from the 2013-2015 survey,^10^ consistent with concerning national trends of students increasingly experiencing these symptoms.^8^ Given that suicide is a leading cause of death for adolescents, our study reinforces the need for pediatric clinicians to screen transgender youth, who are particularly at risk.^8^ Moreover, psychosocial supports, such as mental health and counseling services in school-based health centers and clinical settings for transgender youth, are important. The differential patterns of psychosocial risk and protective factors for BLTY compared with peer groups suggest that BLTY may require approaches in promoting their resilience while accounting for experiences of belonging to racial/ethnic minority groups and being gender diverse.

BLTY includes youth with diverse cultural experiences. Our study grouped Black and Latinx transgender youth as an important first step in documenting and examining their experiences of belonging to racial/ethnic and gender minority groups. Also, our approach of focusing on BLTY is grounded in the poor psychosocial and health outcomes observed in their Black and Latinx adult counterparts.^18,19,23^ Our study reinforces the need to further examine the mental health needs of BLTY and mechanisms that lead to such marked disparities in adulthood using an intersectional stigma lens. Elucidating protective factors among transgender youth will aid in promoting a healthy transition from adolescence to adulthood. Moreover, future studies—both quantitative and qualitative—are needed to examine the nuanced differences among specific race and ethnic minority groups and to determine the culturally specific experiences of intersectionality for these groups.^48^ Furthermore, it is imperative that future research examine BLTY and other transgender youth of color in other socioecological domains within families, peers, and community organizations and to explore how social and environmental factors, such as food insecurity and homelessness, are associated with their mental health. Finally, future research must explore forms of resilience, such as cultural pride, spirituality, and community connectedness, as potential protective factors and as potential areas of focus to promote and support resilience in BLTY. A concerted effort from schools and pediatric medical professionals can potentially address mental health disparities experienced by BLTY through the comprehensive lens of their psychosocial experiences.

### Limitations and Strengths

This study has limitations. First, given that this is an observational study, causality cannot be inferred among peer groups, mental health symptoms, and psychosocial factors. Additionally, this research only focused on past-year depressive symptoms and suicidality; further studies to assess severity and other mental health symptoms, such as anxiety and gender dysphoria, among BLTY are critical. Misclassification was possible given that transgender identity was not measured separately from sexual identity; instead, 1 item queried both sexual identity and gender identity. Sex was also not defined in the survey as birth-assigned sex or distinguished from gender identity. Current best practices recommend assessment of gender identity using a 2-step method querying current gender identity and assigned sex at birth.^39,40,41,42^ The only gender diverse option provided was transgender; youth who identify as nonbinary or gender fluid may not have selected transgender. Without additional gender diverse options, it is not possible to conduct comparisons among youth with different designated sexes at birth or gender identities; this is crucial to address in future studies and population-based survey development. Furthermore, the survey only captured youth in school and may not be generalizable to youth not attending school. Moreover, asking youth to provide attributions for why they were harassed or bullied—eg, because of their race, perceived sexuality, or gender—may create attribution bias. It is difficult, if not impossible, for people to meaningfully separate different aspects of their identities or attribute harassment they experience to a single aspect of their identity. The survey also did not capture information regarding access to gender-affirming medical treatments or parental support, which have been shown to directly affect mental health for transgender youth.^43,44,45^

Despite limitations, our study had numerous strengths. The large, representative probability sample allowed us to make novel comparisons between relevant peer groups. Additionally, the survey assessed important psychosocial factors that allowed comprehensive assessments of BLTY resilience and risk. Finally, this survey was administered in a school setting, an important socioecological domain for youth.^24,25^ Schools are particularly salient targets for culturally based interventions to support BLTY. Organizations such as the Gay, Lesbian, and Straight Education Network recommend intersectional approaches in supporting Black and Latinx sexual minority and gender diverse youth. These approaches include supporting student clubs, such as Gay Straight Alliance, and ethnic/cultural clubs collaborating to address their needs and professional development for school staff that addresses unique experiences of these youth.^46,47^ Pediatric and adolescent medicine clinicians should be involved in school-based efforts to address the unique needs of these youth. For example, they could provide in-service training referral resources for school staff and parent-teacher organizations.

## Conclusions

In this survey study, BLTY and White transgender youth had comparable high rates of mental health symptoms; however, BLTY had disproportionally higher rates than Black and Latinx cisgender youth. The unique pattern of psychosocial risk and protective factors for these mental health symptoms among BLTY should be factored in clinical preventive services and school-based interventions to support them.
